# Reliability and Validity of the Japanese Version of the 12-Item Psychosocial Safety Climate Scale (PSC-12J)

**DOI:** 10.3390/ijerph182412954

**Published:** 2021-12-08

**Authors:** Akiomi Inoue, Hisashi Eguchi, Yuko Kachi, Sarven S. McLinton, Maureen F. Dollard, Akizumi Tsutsumi

**Affiliations:** 1 Institutional Research Center, University of Occupational and Environmental Health, 1-1 Iseigaoka, Yahatanishi-ku, Kitakyushu 807-8555, Japan; 2 Department of Mental Health, Institute of Industrial Ecological Sciences, University of Occupational and Environmental Health, 1-1 Iseigaoka, Yahatanishi-ku, Kitakyushu 807-8555, Japan; eguchi@med.uoeh-u.ac.jp; 3 Department of Public Health, Kitasato University School of Medicine, 1-15-1 Kitazato, Minami-ku, Sagamihara 252-0374, Japan; kachi@med.kitasato-u.ac.jp (Y.K.); akizumi@kitasato-u.ac.jp (A.T.); 4 PSC Global Observatory, Centre for Workplace Excellence, Justice and Society, University of South Australia, Adelaide 5001, Australia; Sarven.McLinton@unisa.edu.au (S.S.M.); Maureen.Dollard@unisa.edu.au (M.F.D.)

**Keywords:** job demands-resources (JD-R) model, primary prevention, psychometric properties, psychosocial risks, work stress

## Abstract

The 12-item psychosocial safety climate scale (PSC-12) has been used extensively in previous research, but its reliability and validity in a Japanese context are still unknown. We examined the psychometrics of the Japanese version of the PSC-12 (PSC-12J). The PSC-12J and scales on the relevant variables were administered to 2200 employees registered with an online survey company. A follow-up survey with 1400 of the respondents was conducted two weeks later. Internal consistency and test–retest reliability were examined via Cronbach’s alpha and Cohen’s weighted kappa coefficients, respectively. Structural, convergent, and known-group validities were examined using confirmatory factor analysis (CFA) and item response theory (IRT) analysis, correlation analysis, and Kruskal–Wallis test, respectively. Cronbach’s alpha and Cohen’s weighted kappa coefficients were 0.97 and 0.53, respectively. CFA based on the four-factor structure established in the previous literature showed an acceptable model fit. IRT analysis showed that each item was an adequate measure of the respondent’s latent trait. Correlations of the PSC-12J with the relevant variables and distribution of scores by demographic characteristics were also observed in the theoretically expected directions, supporting the construct validity of the PSC-12J. Our findings establish the PSC-12J as a reliable and valid measure of the psychosocial safety climate construct in the Japanese context.

## 1. Introduction

In Japan, nearly 60% of employees suffer from stress and other problems at work [1]. Applications for compensation for work-related injuries due to mental disorders have seen more than a tenfold increase over the past two decades [2], and the further dissemination of not only approaches to individuals, but also to organizations via improvements in psychosocial work environment, is required as a measure for mental health among employees [3]. Psychosocial safety climate (PSC) is a dimension of organizational climate and refers to shared employee perceptions regarding “policies, practices, and procedures for the protection of psychological health and safety among employees” [4]. Previous studies in Australia, where PSC was first proposed and developed, have shown that employee perceptions of low PSC were associated with a variety of adverse mental and physical health, as well as safety outcomes, such as psychological distress, emotional exhaustion, circulatory diseases, and occupational injuries [5,6,7]. Given that PSC is an upstream predictor of these outcomes, it is important to establish whether this construct is generalizable to different cultural settings, and to assess it in a variety of national contexts.

PSC is proposed as an organizational factor that stems primarily from management and is an antecedent of task-level or workgroup-level hazards [8]. Therefore, PSC is a precursor to factors covered in previous dominant job stress theories, such as the job demand-resources (JD-R) model [9] and its foundations, i.e., the job demands-control (JD-C)/demand-control-support (DCS) [10,11] and effort–reward imbalance (ERI) models [12].

PSC theory extends pathways inherent in the above-mentioned job stress theories, because it is an antecedent to the job hazards specified in them. In the present study, we use the PSC-extended JD-R model [4] to frame our expectations about how PSC relates to its variables and our decisions in selecting variables for study. In this model, PSC extends the health erosion path of the JD-R model, whereby low PSC increases job demands (e.g., psychological demands and emotional demands), which in turn deteriorate employee mental and physical health (i.e., the health impairment process). PSC extends the motivational path of the JD-R model, whereby high PSC increases the available job resources (e.g., job control, extrinsic reward, and workplace social support), which in turn enhances work engagement (i.e., the motivational process). These extended theoretical paths were supported by empirical studies [4,5,13].

In terms of PSC composition, there are four components: management commitment (MC); management priority (MP); organizational communication (OC); and organizational participation (OP). MC refers to whether senior management is supportive and committed to stress prevention through involvement and commitment. MP refers to whether management prioritizes the psychological health and safety of their employees over productivity goals. OC refers to whether an organization listens to contributions from employees in relation to factors that affect psychological health. OP refers to whether there is a consultation and active participation in health and safety matters from all levels of the organization.

Whilst the first measurement scale that was developed based on the definition of PSC contained 26 items [14], with successive large-scale, population-based research, the scale was systematically reduced to 12 items (three for each subscale). This “PSC-12” [8] was shown to have an acceptable internal consistency (Cronbach’s alpha coefficients, PSC-12: 0.94–0.97; MC: 0.88–0.91; MP: 0.90; OC: 0.76–0.77; and OP: 0.80), structural validity (confirmatory factor analysis (CFA) demonstrated an acceptable model fit and supported the four-factor model), and other aspects of construct validity (mainly convergent validity), such as correlations with other relevant psychosocial work environments and health-related outcomes [5,6,7,8]. Moreover, the PSC-12 was translated into some Asian (e.g., Malay and Chinese) and European languages (e.g., Dutch, French, German, and Swedish), and their reliability and validity, as well as epidemiological findings using the translated scales, were reported [15,16,17,18,19]. On the other hand, in Japan, the concept of PSC is not well known, and thus there are no epidemiological findings on the association of PSC with health among Japanese employees. The concept of PSC, which focuses on “whether management and employees are working together to maintain and promote mental health”, may have a high affinity with Japanese workplaces that are promoting health and productivity management (H&PM), a positive movement that is expected to spread in Japan in the future. Therefore, translating the PSC-12 into Japanese and establishing its psychometrics will be beneficial for research and the practice of occupational health in Japan.

The purpose of the present study was to examine the reliability (i.e., internal consistency and test–retest reliability) and validity (i.e., structural, convergent, and known-group validities) of the Japanese version of the PSC-12 (hereinafter called “PSC-12J”) in a general working population in Japan. The present study was reported in accordance with the Consensus-Based Standards for the Selection of Health Measurement Instruments (COSMIN) Risk of Bias (RoB) checklist (Boxes 3, 4, 6, 7, and 9), which is used to improve the quality of efforts to develop health-related, self-report measurement instruments [20].

## 2. Materials and Methods

### 2.1. Participants

From October to November 2020, an online survey was conducted with registrants of Rakuten Insight, Inc., a private Japanese online survey company. A total of 87,060 people (42,784 men and 44,276 women) were randomly selected from approximately 1.19 million possible registrants (570,000 men and 620,000 women). In the advertisement to the randomly selected audience, participation was incentivized with online shopping points valued at approximately a few US dollars. Responses were on a first-come-first-served basis, and, due to the project budget, the recruitment ceased when the number of respondents reached our target *N* = 2200 (RoB Box 3-3). The 2200 participants answered (1) “I am currently working.” and (2) “I am employed by a company, organization, government office, or self-employed person or private family to earn a salary or wage (including executives).” in the preliminary eligibility screening in order to participate in the main (baseline) survey, which included the PSC-12J and scales on relevant variables. In this baseline survey, the sex ratio was 1:1 and there were an equal number of participants in each age group (20–29, 30–39, 40–49, 50–59, and 60–69 years) due to the ‘stratified sample’ recruitment process.

To further examine the test–retest reliability of the PSC-12J, a follow-up online survey using the PSC-12J was conducted two weeks later, which is the recommended time interval for test–retest reliability (RoB Boxes 6-2 and 7-2) [21]. As with the baseline survey, responses to the follow-up survey were on a first-come-first-served basis and ceased when the number of respondents reached *N* = 1400 (RoB Boxes 6-1, 6-3, 7-1, and 7-3). Because the online survey required all questions to be answered, no participants had missing items.

### 2.2. Measures

#### 2.2.1. PSC-12J

The Japanese translation of the PSC-12 was conducted in collaboration with an Australian team who worked in PSC research (S.S.M. and M.F.D.; S.S.M. is fluent in Japanese) and a Japanese team of experts in job stress research (A.I., H.E., and A.T.). The PSC-12 comprises four subscales (MC, MP, OC, and OP) based on the reflective model, and each subscale is measured with three items (e.g., MC: “In my workplace, senior management acts quickly to correct problems/issues that affect employees’ psychological health”. MP: “Psychological well-being of staff is a priority for this organization”. OC: “There is good communication here about psychological safety issues which affect me”. OP: “Participation and consultation in psychological health and safety occurs with employees, unions, and health and safety representatives in my workplace”.). Each item is measured on a five-point Likert-type response option: 1 = *Strongly disagree*; 2 = *Disagree*; 3 = *Neither agree nor disagree*; 4 = *Agree*; and 5 = *Strongly agree*. The total scores of the PSC-12 (range: 12–60) and its subscales (range: 3–15) are calculated by summing item scores, with higher score indicative of better status.

The translation process of the PSC-12 was based on the International Society for Pharmacoeconomics and Outcomes Research (ISPOR) taskforce guidelines [22]. For the forward translation (from English to Japanese), the Japanese team prepared a draft of the translation and conducted cognitive interviews with occupational health professionals, researchers, and laypersons, asking them to comment on its readability. In parallel, the Japanese team asked the Australian team to make general comments on the draft. After slight amendments to the draft based on comments from cognitive interviews and the Australian team, the amended version was back-translated into English by an independent translator who was blind to the original English version. The back-translated version was reviewed again and harmonized by the Australian team, and the Japanese version was finalized after further slight amendments based on their suggestions. The result was a robust translation of the PSC-12, which in theory presents an accurate reflection of the PSC construct and its four subscales in Japanese.

#### 2.2.2. Relevant Variables (RoB Boxes 9-1 and 9-2)

- Job demands

Based on a previous study on the development of the original PSC-12 [8], we selected (1) psychological demands; (2) physical demands; and (3) emotional demands as the job demands that should show negative relationships with PSC. Scales that measure these constructs were selected from existing well-validated Japanese versions that demonstrated reliable metrics in previous research [23,24,25].

Psychological demands were measured with the 22-item version of the Job Content Questionnaire (JCQ) [23,26]. The JCQ includes a five-item psychological demands scale, measured on a four-point Likert-type scale ranging from 1 = *Strongly disagree* to 4 = *Strongly agree*. The total score (range: 12–48) was calculated according to the JCQ user’s guide [26]. In this sample, Cronbach’s alpha coefficient was 0.65.

Physical demands were measured with the Brief Job Stress Questionnaire (BJSQ) [24]. The BJSQ includes a single-item physical demands scale, “My job requires a lot of physical work.”, measured on a four-point Likert-type scale ranging from 1 = *Not at all* to 4 = *Very much so*.

Emotional demands were measured with the New Brief Job Stress Questionnaire (NBJSQ) [25]. The NBJSQ includes a three-item emotional demands scale, measured on a four-point Likert-type scale ranging from 1 = *Not at all* to 4 = *Definitely*. The total score (range: 3–12) was calculated by summing the scores for each item. In this sample, Cronbach’s alpha coefficient was 0.91.

For these scales, higher scores indicate a more demanding or stressful situation.

- Job resources

For the same reason as mentioned above [8], we selected (1) decision authority; (2) skill discretion; (3) extrinsic reward; (4) supervisor support; and (5) coworker support as job resources which should show positive relationships with PSC. Again, we used existing well-validated Japanese versions of scales that measure these constructs [23,27].

Decision authority, skill discretion, supervisor support, and coworker support were measured with the JCQ [23,26] introduced above. The JCQ includes a three-item decision authority; a six-item skill discretion; a four-item supervisor support; and a four-item coworker support scales, measured on a four-point Likert-type scale ranging from 1 = *Strongly disagree* to 4 = *Strongly agree*. The total scores (range: 12–48 for decision authority and skill discretion; 4–16 for supervisor support and coworker support) were calculated according to the JCQ user’s guide [26]. In this sample, Cronbach’s alpha coefficients were 0.72, 0.67, 0.91, and 0.87 for decision authority, skill discretion, supervisor support, and coworker support, respectively.

Extrinsic reward was measured with the short version of the Effort–Reward Imbalance Questionnaire (Short ERIQ) [27,28]. The Short ERIQ includes a seven-item extrinsic reward scale measured on a four-point Likert-type scale ranging from 1 = *Strongly disagree* to 4 = *Strongly agree*. Of the seven items, three items indicate an adverse condition, and the other four do not; the scoring for the former adverse items is reversed such that the overall scale measured ‘positive’ extrinsic rewards. The total score (range: 7–28) was calculated by summing the scores for each item. In this sample, Cronbach’s alpha coefficient was 0.68.

For these scales, higher scores indicate more resources at work.

- Outcomes

For outcome measures which should show significant relationships with PSC, we focused not only on negative measures, but also positive measures. For the negative measures, we selected (1) psychological distress and (2) emotional exhaustion, whereas, for the positive measures, we selected (3) self-rated health; (4) work engagement; and (5) job satisfaction [8]. Established Japanese versions of scales were used to measure these constructs [24,29,30,31].

Psychological distress was measured with the K6 scale [29,32]. The K6 scale comprises six items measuring the levels of psychological distress on a five-point Likert-type scale ranging from 0 = *None of the time* to 4 = *All of the time*. The total score (range: 0–24) was calculated by summing the scores for each item. In this sample, Cronbach’s alpha coefficient was 0.94.

Emotional exhaustion was measured with the Burnout Assessment Tool (BAT) [30,33]. The BAT includes an eight-item emotional exhaustion scale, measured on a five-point Likert-type scale ranging from 1 = *Never* to 5 = *Always*. The total score (range: 1–5) was calculated by averaging the scores for each item. In this sample, Cronbach’s alpha coefficient was 0.94.

Self-rated health was measured with a single-item question “What is your current state of health?”, on a five-point Likert-type scale ranging from 1 = *Not good* to 5 = *Good*.

Work engagement was measured with the nine-item version of the Utrecht Work Engagement Scale (UWES-9) [31,34]. The UWES-9 comprises nine items measuring the levels of work engagement on a seven-point Likert-type scale ranging from 0 = *Never* to 6 = *Always (everyday)*. The total score (range: 0–6) was calculated by averaging the scores for each item. In this sample, Cronbach’s alpha coefficient was 0.96.

Job satisfaction was measured with the BJSQ [24] introduced above. The BJSQ includes a single-item job satisfaction scale “I am satisfied with my job.” measured on a four-point Likert-type scale ranging from 1 = *Dissatisfied* to 4 = *Satisfied*.

For the negative measures (i.e., psychological distress and emotional exhaustion), higher scores indicate higher levels of distress or exhaustion, whereas, for the positive measures (i.e., self-rated health, work engagement, and job satisfaction), higher scores indicate a better condition.

#### 2.2.3. Demographic Characteristics

For demographic characteristics, we measured sex, age group, education, and occupation. Information regarding sex and age group (20–29, 30–39, 40–49, 50–59, and 60–69 years) was obtained from the participant data that were registered to the online survey company. Information on education and occupation was obtained from the online survey. Education was classified into four groups: graduate school, college, junior college, and high school or junior high school. Occupation was also classified into four groups: manager, non-manual, manual, and others (RoB Box 9-5).

### 2.3. Statistical Analysis

Because the Kolmogorov–Smirnov test did not show normality in the distribution of the total scores of the PSC-12J and its subscales (*p* < 0.001), nonparametric tests were mainly used to test the hypotheses. We first calculated the means, standard deviations (*SD*s), medians, and quartile deviations (*QD*s) for the total scores of the PSC-12J and its subscales.

Then, to examine the internal consistency and test–retest reliability, Cronbach’s alpha coefficient (RoB Box 4-2) and Cohen’s weighted kappa coefficient with linear weight (RoB Boxes 6-5 to 6-7) were calculated, respectively. As parametric statistics for test–retest reliability, intraclass correlation coefficient (ICC [1,1]) (RoB Box 6-4) and standard error of measurement (SEM) (RoB Box 7-4) were also calculated. For the calculation of the Cohen’s weighted kappa coefficient, ICC, and SEM, we used data from 1400 participants who responded to the follow-up survey.

Furthermore, to examine the structural validity, CFA was conducted, which allowed us to assess the goodness of fit for the structure of PSC established in other literature (i.e., four-factor structure of MC, MP, OC, and OP) (RoB Box 3-1). Model fit was assessed using a combination of fit indices including the goodness of fit index (GFI), the adjusted goodness of fit index (AGFI), the comparative fit index (CFI), the Tucker–Lewis index (TLI), and the root mean square error of approximation (RMSEA). The acceptability of model fit was judged by the following criteria: GFI, AGFI, CFI, and TLI > 0.90 and RMSEA < 0.08 [35]. Then, an item response theory (IRT) analysis with the generalized partial credit model [36] (RoB Box 3-2) was conducted for each subscale to estimate discrimination (*a*) and difficulty (*b*/threshold) for each item. If the discrimination was *a* > 0.50 and the difficulty was |*b*| < 4.00, the item was judged to be an adequate measure of the respondent’s latent trait [37,38].

Furthermore, as a hypothesis test for convergent validity, Spearman’s rank correlation coefficients were calculated between scores on the PSC-12J and its subscales and relevant variables introduced earlier (RoB Box 9-3). Following Cohen [39], we describe effects as small (0.10), medium (0.30), and large (0.50). Based on findings from a previous study [8], it was hypothesized that the PSC-12J (and its subscales) would show small-to-medium negative correlations with psychological demands and emotional demands (−0.30 < *ρ* < −0.10). It was also hypothesized that the PSC-12J would show a negative correlation with physical demands, but that it would be quite small (−0.10 < *ρ* < 0) since PSC focuses mainly on psychosocial safety rather than physical safety. For job resources, it was hypothesized that the PSC-12J would show small-to-medium positive correlations regarding decision authority, skill discretion, and coworker support (0.10 < *ρ* < 0.30), which are individual-level or interpersonal-level resources; and medium-to-large positive correlations with extrinsic reward and supervisor support (*ρ* > 0.30), which are more closely related to management behavior. For outcome measures, it was hypothesized that the PSC-12J would show small-to-medium negative correlations with poor psychological-health-related measures (psychological distress and emotional exhaustion) (−0.30 < *ρ* < −0.10) and a positive correlation with self-rated health (0.10 < *ρ* < 0.30); and medium-to-large positive correlations with work motivation (work engagement and job satisfaction) (*ρ* > 0.30) [8].

Lastly, the Kruskal–Wallis test was conducted as another hypothesis test for known-group validity to compare the mean ranks of the PSC-12J and its subscales on each demographic characteristic (RoB Box 9-6). Japanese companies have a male-oriented corporate culture [40], and people with higher education levels are more likely to have a job with a better (or more resourceful) work environment [41]. Since people in managerial positions are directly involved in the construction of the PSC, we hypothesized that the mean ranks of the PSC-12J would be higher among men, those with a higher education, and managers.

The level of significance was 0.05 (two-tailed). All the analyses were conducted using the IBM SPSS Statistics Version 27.0, Amos Version 27.0, and Stata 14.2.

## 3. Results

Table 1 shows the detailed characteristics of the participants at the baseline and follow-up surveys. As noted earlier, the sex ratio and the number of participants in each age group were equal. For education and occupation, college graduates and non-manual employees had the highest proportions. There were no differences in the distribution of demographic characteristics between the baseline and follow-up surveys (RoB Box 9-5).

Table 2 shows means, *SD*s, medians, *QD*s, Cronbach’s alpha coefficients, Cohen’s weighted kappa coefficients, ICCs, and SEM for the PSC-12J and its subscales (RoB boxes 4-1, 4-2, 6-4, 6-6, and 7-4). Cronbach’s alpha coefficients were high for all subscales, suggesting good internal consistency. Furthermore, Cohen’s weighted kappa coefficients and ICCs showed a moderate test–retest reliability.

Table 3 shows the results of the CFA and IRT analysis assuming the four-factor structure of MC, MP, OC, and OP. For CFA, standardized factor loadings were greater than 0.85 and all significant for each factor. Furthermore, covariances among factors were greater than 0.80 and all significant. Fit indices showed that the originally hypothesized four-factor structure yielded an acceptable model fit (GFI = 0.97; AGFI = 0.94; CFI = 0.99; TLI = 0.98; and RMSEA = 0.06) (RoB Box 3-1). For IRT analysis, all items met the criteria of *a* > 0.50 for discrimination and |*b*| < 4.00 for difficulty, indicating that each item adequately measured the respondent’s latent trait. The standard errors of the theta (*SE*(*θ*)) for each subscale were all 0.020 (RoB Box 4-4).

Table 4 shows Spearman’s rank correlation coefficients of the PSC-12J (and its subscales) with other variables of interest. For job demands, psychological demands and emotional demands showed small-to-medium negative correlations with the PSC-12J. Physical demands also showed a negative correlation with the PSC-12J whilst it was relatively small. For job resources, decision authority and skill discretion showed small-to-medium positive correlations; extrinsic reward, supervisor support, and coworker support showed medium-to-large positive correlations with the PSC-12J. For negative outcome measures, psychological distress and emotional exhaustion showed small-to-medium negative correlations with the PSC-12J. For positive outcome measures, self-rated health showed small-to-medium positive correlations; work engagement and job satisfaction showed medium-to-large positive correlations with the PSC-12J. Similar tendencies were observed for the subscales (RoB Box 9-3).

Table 5 shows the comparison of the mean ranks of the PSC-12J and its subscales by demographic characteristics. The results of the Kruskal–Wallis test showed that there were significant group differences in the mean ranks of the PSC-12J for sex, education, and occupation. More specifically, men, those with higher education (i.e., college graduate or higher), and managers had higher mean ranks compared to the counterparts. On the other hand, a significant group difference in the mean ranks of the PSC-12J was not observed for age. With two exceptions (i.e., a significant group difference in the mean ranks of the MC and OC for age), similar tendencies were observed for subscales (RoB Box 9-6).

In summary, the PSC-12J showed excellent psychometric qualities, suggesting that the Japanese translation is an accurate and reliable reflection of the PSC construct in a Japanese context.

## 4. Discussion

In the present study, the English version of the PSC-12 was translated into Japanese, and the reliability and validity of the PSC-12J were examined in a general working population in Japan. The PSC-12J and its subscales showed a high internal consistency and moderate test–retest reliability. The factor structure, correlations with other relevant psychosocial work environments and health-related outcomes, and the distribution of scores by demographic characteristics were also observed to be in the theoretically expected directions.

Specifically, the PSC-12J and its subscales had Cronbach’s alpha coefficients of >0.90, indicating a high internal consistency. These coefficients are similar to or higher than those reported in the previous study in Australia [8]; therefore, the new PSC-12J seems to have a level of reliability that is comparable to the original English version. Furthermore, it showed a moderate test–retest reliability (Cohen’s weighted kappa = 0.53 and ICC = 0.69), which was similar to the result of a previous study reported in Australia with a 12-month interval [42]. There is ongoing discussion in the literature about how stable climate factors should be expected to act over time, with some experts suggesting that a fluctuation can be expected, whilst others claim that climate endures in an organization (especially when disenfranchised employees or toxic workplace behaviors persist). In the online survey conducted in the present study, PSC was asked first in both the baseline and follow-up surveys. In addition, considering that the interval between the surveys was only two weeks, the moderate test–retest reliability observed in the present study is unlikely to be due to a lack of attention when answering the PSC-12J, but rather due to a certain factor causing PSC to fluctuate between the surveys. For example, some of the managers who participated in the present study may have been inspired by the PSC-12J items and started working on improving their PSC immediately after the baseline survey. Another possibility is that some participants may have become more aware of psychosocial risks and hazards due to answering questions about PSC in the baseline survey, and their response patterns to the PSC-12J may have changed in the follow-up survey. There is also a dearth of research on PSC over very short time intervals, so the potential for PSC to fluctuate over the course of two weeks is still unknown. Together, all of these factors may have affected the test–retest reliability. Therefore, whilst our findings suggest that the PSC-12J has a certain level of reproducibility, the temporal stability of PSC needs further detailed discussions, including international comparisons.

The results of CFA based on the originally assumed four-factor structure showed an acceptable model fit. The fit indices are similar to or better than those reported in the previous study in Australia [8], indicating that the PSC-12J has a similar factor structure to the original English version and they are comparable to each other. Furthermore, the IRT analysis showed that all items had a sufficient discrimination and appropriate difficulty, suggesting that the items comprising the subscales of the PSC-12J are sensitive in identifying PSC and are suitable for measuring PSC in Japanese workplaces.

The results of the correlations of the PSC-12J and its subscales with the relevant psychosocial work environment and health-related outcomes were generally in line with the hypotheses and consistent with the previous study in Australia [8]. The only exception was that in our study, the correlation coefficient of the PSC-12J with coworker support was much greater than in the previous study in Australia (0.45 and 0.17, respectively). Compared to Western countries, Japanese workplaces are characterized by not only a more collectivistic approach among coworkers, but also a hierarchy oriented between senpai and kōhai (which refers to the social dynamics of a senior–junior relationship) [43]; therefore, policies that are established and implemented to protect psychological health and safety among employees may be more likely to have an impact on the relationships among coworkers in Japanese workplaces compared to Australian (or similar Western) workplaces. Further cross-cultural research on the association of PSC with various kinds of relevant variables is needed.

The results of the Kruskal–Wallis test showed that there were significant group differences in the mean ranks of the PSC-12J for sex, education, and occupation. Particularly, men, those with higher education, and managers had higher mean ranks compared to their counterparts. Although known-group validity was not examined or reported in the previous study in Australia [8], our findings are reasonable since Japanese workplaces have a male-oriented culture (i.e., the participation of women in the important decision-making process has yet to be promoted) [40]; people with higher education are reported to be more likely to have a job in a more resourceful work environment [41]; and people in managerial positions are directly involved in the construction of the PSC. People with such demographic characteristics may have been more likely to more highly rate the PSC of their own workplace. Our findings suggest that the Japanese version of the PSC-12 has a certain level of known-group validity.

Possible limitations of the present study should be considered. First, the present study was conducted among registrants of one particular private online survey company. Our study sample may be more likely to be healthy individuals who are satisfied with their circumstances (i.e., less likely to be overworked, bullied, depressed, or dissatisfied with their jobs). On the other hand, it is also possible that our study sample included individuals who were in need and participated in the survey to obtain incentives. Therefore, the mean scores of the PSC-12J and its subscales shown in Table 2 are only preliminary and generalizability should be made with caution. Further research using recruitment strategies that account for non-Internet users should be conducted to calculate mean scores that are more generalizable to Japan as a whole. Second, in the present study, the PSC-12J showed a high internal consistency, but the Cronbach’s alpha coefficient was over 0.90, indicating that there may be a redundancy in the scale items. Third, some of the relevant variables used to examine convergent validity (e.g., physical demands) were measured with single-item scales. Because single-item scales have a low content validity and sensitivity, and internal consistency cannot be calculated, and it is possible that the true association with the PSC-12J was masked. In the future, it is necessary to confirm whether the association found in the present study can be replicated using a multi-item scale with a confirmed reliability and validity. Lastly, some important properties of the scale, such as cross-cultural validity (RoB Box 5), criterion validity (RoB Box 8), and responsiveness (RoB Box 10), were not examined in the present study, and such properties should be examined in the future.

Nevertheless, we cannot understate the notable strengths of the present study. First, we base our analysis on a very large sample of the general community (stratified to account for sex and age brackets), and used matched data for the same people over time. Next, the reliability and validity results were based on extensive testing via different metrics; therefore, we could be confident the PSC-12J tapped into the same fundamental construct that was measured in Western countries. Lastly, the thorough back-translation process and the IRT analysis were a commitment that very few research measures engaged in when translating a scale into a different language, a first for PSC-12 translation, and we can be confident that the PSC-12J is a robust reflection of the psychosocial safety climate in a Japanese context, accounting for the subtleties in language and culture.

Future research could explore the psychometrics of the PSC-12J short form; since the internal consistency of the PSC-12J was so high, this implies some redundancy. For example, a single-item measure of each of the four domains (i.e., a PSC-4J) could present a reliable way of measuring PSC whilst balancing the practicalities of reducing participant burden [42,44]. Given that the PSC construct refers to a shared perception, the reliability and validity of the measure could be explored further using multilevel modelling and CFA. Grouping data by organization was not possible in the current population study across numerous organizations.

## 5. Conclusions

Our findings provided evidence that the PSC-12J is reliable, valid, and a comparable measure to the established English version. Although more detailed validity needs to be examined in the future, we hope that the PSC-12J will be a useful instrument for assessing PSC in Japanese workplaces, and by extension can further lead the charge for primary prevention and intervention research [45] for improved psychological health among Japanese employees.

## Figures and Tables

**Table 1:** Demographic characteristics of employees who participated in the present study.

Demographic Characteristics	Baseline	Follow-Up ^1^
	*n* (%)	*n* (%)
**Sex**		
Men	1100 (50.0)	700 (50.0)
Women	1100 (50.0)	700 (50.0)
**Age**		
20–29 years old	440 (20.0)	281 (20.1)
30–39 years old	440 (20.0)	281 (20.1)
40–49 years old	440 (20.0)	279 (19.9)
50–59 years old	440 (20.0)	281 (20.1)
60–69 years old	440 (20.0)	278 (19.9)
**Education**		
Graduate school	126 (5.73)	87 (6.21)
College	1054 (47.9)	655 (46.8)
Junior college	491 (22.3)	299 (21.4)
High school/Junior high school	529 (24.0)	359 (25.6)
**Occupation**		
Manager	249 (11.3)	158 (11.3)
Non-manual	1508 (68.5)	959 (68.5)
Manual	294 (13.4)	188 (13.4)
Others	149 (6.77)	95 (6.79)

^1^ Follow-up sample was used only to examine the test–retest reliability.

**Table 2:** Means, standard deviations (*SD*s), medians, quartile deviations (*QD*s), Cronbach’s *α* coefficients, Cohen’s weighted *κ* coefficients, intraclass correlation coefficients (ICCs), and standard error of measurement (SEM) for the PSC-12J and its subscales (*N* = 2200 for mean, *SD*, median, *QD*, and Cronbach’s *α* coefficient; *N* = 1400 for Cohen’s weighted *κ* coefficient, ICC, and SEM).

Scale (Score Range)	Mean	*SD*	Median	*QD*	Cronbach’s *α*	Cohen’s Weighted *κ* (95% *CI*) ^1^	ICC (95% *CI*) ^1^	SEM (95% *CI*) ^1^
PSC-12J (total) (12–60)	34.8	11.4	36.0	7.5	0.97	0.53 (0.50–0.56)	0.69 (0.67–0.72)	6.31 (6.03–6.59)
**PSC-12J subscales**								
Management commitment (3–15)	8.71	3.07	9.0	2.5	0.93	0.48 (0.45–0.52)	0.63 (0.60–0.66)	1.84 (1.76–1.92)
Management priority (3–15)	8.77	3.17	9.0	2.5	0.94	0.49 (0.46–0.52)	0.64 (0.61–0.67)	1.88 (1.80–1.96)
Organizational communication (3–15)	8.77	3.01	9.0	2.0	0.91	0.49 (0.46–0.52)	0.65 (0.62–0.68)	1.76 (1.69–1.84)
Organizational participation (3–15)	8.52	3.07	9.0	2.0	0.92	0.49 (0.46–0.52)	0.64 (0.61–0.67)	1.81 (1.73–1.89)

^1^ *CI* = confidence interval.

**Table 3:** Confirmatory factor analysis (standardized factor loading of items in the four-factor structure and covariance among factors) and item response theory analysis (discrimination and difficulty of items and standard error of the theta (*SE*(*θ*)) in each subscale) of the PSC-12J (*N* = 2200).

	Confirmatory Factor analysis (CFA) ^1^				Item Response Theory (IRT) Analysis ^2^					
	Standardized Factor Loading				Discrimination (*a*)	Difficulty (*b*/Threshold)				*SE(θ*)
	MC	MP	OC	OP		*b*_1_ (2 vs. 1)	*b*_2_ (3 vs. 2)	*b*_3_ (4 vs. 3)	*b*_4_ (5 vs. 4)	
**Management commitment (MC)**										0.020
Q1. MC1 (Act quickly)	0.90				4.31	−1.17	−0.44	0.49	1.55	
Q2. MC2 (Act decisively)	0.93				7.62	−1.10	−0.40	0.52	1.55	
Q3. MC3 (Show support)	0.90				3.80	−1.09	−0.38	0.64	1.63	
**Management priority (MP)**										0.020
Q4. MP1 (Priority)		0.88			3.13	−1.05	−0.39	0.63	1.65	
Q5. MP2 (Importance)		0.93			6.17	−1.07	−0.38	0.43	1.34	
Q6. MP3 (As important as productivity)		0.94			8.98	−1.00	−0.37	0.49	1.35	
**Organizational communication (OC)**										0.020
Q7. OC1 (Good communication)			0.86		2.96	−1.27	−0.61	0.44	1.66	
Q8. OC2 (Information available)			0.88		4.15	−1.04	−0.39	0.63	1.59	
Q9. OC3 (Contribution being listened to)			0.90		3.51	−1.20	−0.54	0.55	1.67	
**Organizational participation (OP)**										0.020
Q10. OP1 (Actual participation)				0.86	3.12	−1.01	−0.41	0.68	1.61	
Q11.OP2 (Participation being encouraged)				0.91	4.91	−1.12	−0.45	0.52	1.57	
Q12. OP3 (Prevention involves all levels)				0.88	3.27	−0.97	−0.35	0.76	1.72	
**Covariance**										
Management priority (MP)	0.95									
Organizational communication (OC)	0.80	0.87								
Organizational participation (OP)	0.81	0.89	0.85							

^1^ For the CFA, all standardized factor loadings with numbers listed and covariances were significant at the *p* < 0.01 level. A blank indicates that there was no path from a factor to an item (i.e., zero factor loading), as hypothetically defined in the model. ^2^ For the IRT analysis, generalized partial credit model was used.

**Table 4:** Spearman’s rank correlation coefficients of the PSC-12J and its subscales with other relevant variables (*N* = 2200) ^1^.

	PSC-12J (Total)	PSC-12J Subscales			
		MC	MP	OC	OP
**PSC-12J subscales**					
Management commitment (MC)	0.91				
Management priority (MP)	0.92	0.85			
Organizational communication (OC)	0.90	0.76	0.77		
Organizational participation (OP)	0.90	0.76	0.78	0.80	
**Relevant variables (job demands and resources)**					
Psychological demands	−0.14	−0.12	−0.16	−0.13	−0.13
Physical demands	−0.09	−0.07	−0.10	−0.07	−0.08
Emotional demands	−0.21	−0.20	−0.20	−0.20	−0.19
Decision authority	0.25	0.23	0.25	0.25	0.23
Skill discretion	0.27	0.23	0.24	0.25	0.27
Extrinsic reward	0.47	0.41	0.43	0.47	0.42
Supervisor support	0.58	0.50	0.52	0.61	0.51
Coworker support	0.45	0.40	0.41	0.46	0.40
**Relevant variables (outcomes)**					
Psychological distress	−0.25	−0.22	−0.25	−0.24	−0.20
Emotional exhaustion	−0.24	−0.21	−0.24	−0.22	−0.21
Self-rated health	0.21	0.19	0.19	0.22	0.18
Work engagement	0.38	0.34	0.35	0.38	0.34
Job satisfaction	0.38	0.34	0.35	0.39	0.33

^1^ All coefficients are significant at the *p* < 0.01 level.

**Table 5:** Comparison of the mean ranks of the PSC-12J and its subscales by demographic characteristics (Kruskal–Wallis test) (*N* = 2200).

	PSC-12J (Total)		PSC-12J Subscales ^1^							
			MC		MP		OC		OP	
	Mean Rank	*p* Value	Mean Rank	*p* Value	Mean Rank	*p* Value	Mean Rank	*p* Value	Mean Rank	*p* Value
**Sex**		0.005		0.034		0.028		0.028		<0.001
Men (*n* = 1100)	1138.15		1128.93		1129.91		1129.99		1151.24	
Women (*n* = 1100)	1062.85		1072.07		1071.09		1071.01		1049.76	
**Age**		0.062		0.046		0.054		0.024		0.141
20–29 years old (*n* = 440)	1134.12		1131.88		1125.45		1155.62		1135.65	
30–39 years old (*n* = 440)	1083.18		1077.77		1062.04		1114.42		1086.29	
40–49 years old (*n* = 440)	1034.81		1037.57		1049.41		1021.88		1040.34	
50–59 years old (*n* = 440)	1100.43		1098.19		1105.05		1087.43		1107.50	
60–69 years old (*n* = 440)	1149.96		1157.09		1160.55		1123.15		1132.72	
**Education**		<0.001		<0.001		<0.001		<0.001		<0.001
Graduate school (*n* = 126)	1142.73		1154.60		1133.73		1129.81		1172.40	
College (*n* = 1054)	1174.23		1153.43		1164.36		1174.79		1168.28	
Junior college (*n* = 491)	1033.29		1051.75		1057.24		1034.70		1017.29	
High school/ Junior high school (*n* = 529)	1005.92		1027.39		1005.50		1006.58		1025.56	
**Occupation**		<0.001		<0.001		<0.001		<0.001		<0.001
Manager (*n* = 249)	1343.94		1346.22		1351.31		1288.39		1314.60	
Non-manual (*n* = 1508)	1086.56		1080.60		1087.17		1091.65		1089.72	
Manual (*n* = 294)	1018.21		1021.67		1006.18		1042.48		1031.85	
Others (*n* = 149)	997.16		1046.85		1002.35		990.60		987.33	

^1^ MC = management commitment; MP = management priority; OC = organizational communication; OP = organizational participation.

## Data Availability

The data presented in this study are not publicly available but are available from the corresponding author on reasonable request.
